# Epidemiology of Nontuberculous Mycobacterial Lung Disease and Tuberculosis, Hawaii, USA

**DOI:** 10.3201/eid2303.161827

**Published:** 2017-03

**Authors:** Jennifer Adjemian, Timothy B. Frankland, Yihe G. Daida, Jennifer R. Honda, Kenneth N. Olivier, Adrian Zelazny, Stacey Honda, D. Rebecca Prevots

**Affiliations:** United States Public Health Service, Commissioned Corps, Rockville, Maryland, USA (J. Adjemian);; National Institute of Allergy and Infectious Diseases, Bethesda, Maryland, USA (J. Adjemian, A. Zelazny, D.R. Prevots);; Kaiser Permanente, Honolulu, Hawaii, USA (T.B. Frankland, Y.G. Daida, S. Honda);; University of Colorado Anschutz Medical Campus, Aurora, Colorado, USA (J.R. Honda);; National Jewish Health, Denver, Colorado, USA (J.R. Honda);; National Heart, Lung, and Blood Institute, Bethesda (K.N. Olivier)

**Keywords:** pulmonary, nontuberculous mycobacteria, bacterial infections, tuberculosis and other mycobacteria, TB, epidemiology, Hawai’i, Hawaii, United States

## Abstract

Previous studies found Hawaiians and Asian-Americans/Pacific Islanders to be independently at increased risk for nontuberculous mycobacterial pulmonary disease (NTMPD) and tuberculosis (TB). To better understand NTM infection and TB risk patterns in Hawaii, USA, we evaluated data on a cohort of patients in Hawaii for 2005–2013. Period prevalence of NTMPD was highest among Japanese, Chinese, and Vietnamese patients (>300/100,000 persons) and lowest among Native Hawaiians and Other Pacific Islanders (50/100,000). Japanese patients were twice as likely as all other racial/ethnic groups to have *Mycobacterium abscessus* isolated (adjusted odds ratio 2.0, 95% CI 1.2–3.2) but were not at increased risk for infection with other mycobacteria species. In contrast, incidence of TB was stable and was lowest among Japanese patients (no cases) and highest among Filipino, Korean, and Vietnamese patients (>50/100,000). Substantial differences exist in the epidemiology of NTMPD by race/ethnicity, suggesting behavioral and biologic factors that affect disease susceptibility.

The incidence of nontuberculous mycobacterial pulmonary disease (NTMPD) is increasing in North America and many parts of the world (*1*–*7*), whereas the incidence of tuberculosis (TB), caused by *Mycobacterium tuberculosis*, has decreased in industrialized countries during the same period (*8*,*9*). However, population-based data are lacking from areas with high incidence of NTMPD and TB.

In 2012, the first-ever US nationwide analysis on the prevalence of NTMPD among older adults found that Hawaii had the highest prevalence of any state, 4 times the national average (1). Another recent study identified Hawaii as having the highest age-adjusted mortality rate from NTMPD (*10*). In these reports, persons identified as Asian American/Pacific Islander were at increased risk for NTMPD, independent of geographic area of residence (*1*). Similarly, Hawaii has a TB incidence greater than the national average (*11*), and among Native Hawaiians and Other Pacific Islanders (NHOPI), this rate is 13 times higher than among non-Hispanic whites. Moreover, 67% of all TB patients and 96% of Asians with TB are foreign-born (*12*); because Hawaii has a large population of foreign-born Asians, it is disproportionately affected (*13*).

The high risk for NTMPD (*1**,**14*) and TB (*11**,**12*) in Hawaii enables us to estimate their relative prevalence in the same population and identify distinct and overlapping risk factors in subpopulations. Because Hawaii has the highest proportion of Asian and NHOPI residents of any state (41% and 9%, respectively) (*15*), this population provides an opportunity to better describe the epidemiology of these diseases in a high-risk setting.

## Materials and Methods

### Study Population

We extracted electronic medical record data on Kaiser Permanente Hawaii (KPH) patients enrolled for >9 months during 2005–2013. We queried databases for all patients with acid-fast bacilli (AFB) smears or mycobacterial cultures performed on respiratory specimens. For each specimen tested, we extracted collection date, body site of collection, AFB smear results, and mycobacterial culture results, including species identified if positive. For patients tested for mycobacteria, we extracted data on selected co-morbidities using codes from the International Classification of Diseases, Ninth Revision (ICD-9). We extracted data on age, sex, self-identified race/ethnicity, and residential zip code for all patients. Racial/ethnic data are based on self-report at enrollment; patients could select >1 of 28 options, including 23 Asian and Pacific Islander subgroups. This study was approved by the National Institutes of Health Office for Human Subjects Research and the KPH Institutional Review Board.

### Laboratory Analysis

We used standard laboratory procedures in a Clinical Laboratory Improvement Amendments–certified laboratory for mycobacterial testing by AFB smear and culture. We used commercially available probes at KPH for identification of *M. tuberculosis* complex and *M. avium* complex (MAC); we sent other isolates to Associated Regional and University Pathologists Laboratories for further species identification by PCR or sequence-based identification. Laboratory methods were consistent across the entire study period. We recorded all mycobacteria species identified; *M. gordonae* was considered nonpathogenic and excluded from case definitions (*16*).

### Case Definitions

We defined 3 case groups. The nontuberculous mycobacteria (NTM) isolation group comprised patients with >1 pathogenic pulmonary NTM species identified; the NTMPD group comprised patients with >2 positive cultures for pathogenic NTM identified; and the TB group comprised patients with >1 pulmonary *M. tuberculosis* isolate, regardless of their NTM isolation status.

### Data Analysis

We calculated annual and overall period prevalence for each case group by using the KPH source population as the denominator. We stratified all estimates by age group, sex, race/ethnicity, and island (for those residing in a single zip code throughout the study). We used Poisson regression models with allowance for overdispersion and an offset given by the log of the KPH population to calculate the annual percentage change in prevalence over time. We used χ^2^ and Student *t* tests to test for differences in case frequencies by demographic factors and co-morbidities. We assessed associations with NTM infection and TB in logistic regression models by using a Firth penalized likelihood option to reduce small-sample bias, where all smear-positive patients were compared with patients not identified as positive. For models evaluating demographic factors, the negative comparison group included those testing negative and those not tested, assuming they were negative. For models evaluating co-morbidities, the negative comparison group included only those testing negative for mycobacteria. To better assess the independent effect of race/ethnicity, we limited regression models to patients with a single racial/ethnic group identified. We adjusted all models for sex, age group, and years present in the KPH database (range 1–9 years). We further adjusted models identifying notable demographic variables by co-morbidities that might be associated with smoking (i.e., chronic obstructive pulmonary disease [COPD], as determined by ICD-9 codes documented for emphysema, obstructive chronic bronchitis, or chronic airway obstruction), for those with co-morbidity data available. We conducted analyses by using SAS version 9.3 (SAS Institute, Inc., Cary, NC, USA) and calculated adjusted odds ratios (aORs) and 95% CIs.

## Results

**Figure 1:**
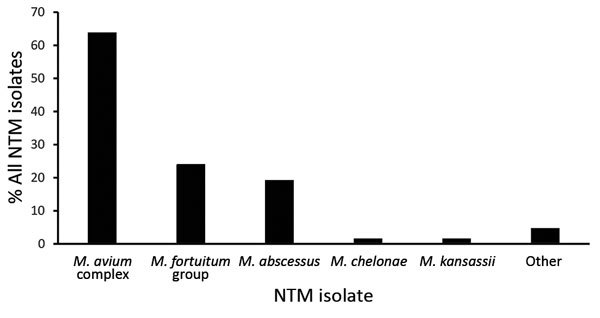
Occurrence of nontuberculous mycobacteria species identified from pulmonary specimens obtained among a cohort of Kaiser Permanente Hawaii patients, Hawaii, 2005–2013. Other pathogenic nontuberculous mycobacteria species identified (n = 21) were *Mycobacterium flavescens, M. immunogenum, M. mucogenicum, M. neoaurum, M. scrofulaceum*, *M. simiae*, and undifferentiated *M. chelonae/abscessus*. NTM, nontuberculous mycobacteria.

During 2005–2013, a total of 373,168 patients were enrolled in KPH, representing nearly one third of the Hawaii population (*15*); the demographic distribution of our study population was similar to that for the state, with slightly more white patients (Table 1) (*15**,**17*). Of the patient total, 2,197 (0.6%) had >1 mycobacterial culture performed on a respiratory specimen; 1,086 (49%) of those had only 1 culture performed (range 1–29 cultures/patient). Of patients who had culture performed, 455 (21%) had pathogenic NTM isolated: 201 (44%) had 1 positive culture, and 254 (56%) had >2 positive cultures (NTMPD cases) (Table 1). The most frequently isolated species were MAC (n = 290; 64%), *M. fortuitum* group (n = 109; 24%), and *M. abscessus* (n = 87; 19%) (Figure 1); 91 (20%) patients had >1 NTM species identified. A higher proportion of patients with *M. abscessus* isolated (30%) were positive for >2 years in the database, compared with those with MAC (16%) or *M. fortuitum* group (6%).

**Table 1:** Demographic characteristics of study population for investigation of mycobacterial infection prevalence, by mycobacterial testing status and culture result, Hawaii, 2005–2013*

Characteristic	KPH population	NTM isolated			TB isolated	Culture negative
		Total	1 positive culture	>2 positive cultures		
Total	373,168 (100)	455 (0.1)	201 (0.05)	254 (0.07)	40 (0.01)	1,707 (0.5)
Female sex	184,292 (49)	245 (54)	100 (50)	145 (57)	12 (30)	797 (47)
Age, mean ± SD, y	36 ± 22	66 ± 16	65.2 ± 6.3	66 ± 16	55 ± 16	62 ± 17
Island of residence†						
Oahu	196,391 (69)	300 (79)	135 (81)	165 (78)	21 (58)	1,061 (78)
Maui	61,427 (21)	56 (15)	22 (13)	34 (16)	13 (36)	220 (16)
The Big Island	26,052 (9)	22 (6)	10 (6)	12 (6)	2 (6)	74 (5)
Kauai	2,452 (1)	0	0	0	0	0
Years in KPH						
1	72,661 (19)	12 (3)	6 (3)	6 (2)	3 (8)	89 (5)
2–4	118,403 (32)	59 (13)	32 (16)	27 (11)	10 (25)	321 (19)
>5	182,104 (49)	384 (84)	163 (81)	221 (87)	27 (68)	1,297 (76)
Race/ethnicity‡						
White	124,966 (43)	172 (38)	76 (38)	96 (38)	4 (10)	685 (40)
Black	6,260 (2)	2 (0.4)	2 (1)	0	0	31 (2)
NHOPI	90,785 (24)	81 (18)	46 (23)	35 (14)	5 (13)	415 (24)
Asian	142,931 (38)	265 (58)	110 (55)	155 (61)	30 (75)	908 (53)
Filipino	60,314 (21)	90 (20)	34 (17)	56 (22)	22 (55)	306 (18)
Japanese	38,571 (13)	92 (20)	40 (20)	52 (20)	0	325 (19)
Chinese	23,932 (8)	50 (11)	20 (10)	30 (12)	2 (5)	190 (9)
Korean	5,967 (2)	19 (4)	9 (4)	10 (4)	2 (5)	39 (2)
Vietnamese	2,030 (0.7)	5 (1)	2 (1)	3 (1)	1 (3)	13 (0.8)
Other	22,801 (8)	20 (4)	9 (4)	11 (4)	3 (8)	83 (5)
>1 Race/ethnicity	105,159 (36)	111 (24)	58 (29)	53 (21)	6 (15)	476 (28)

*Values are no. (%) patients unless otherwise indicated. A total of 2,197 patients had pulmonary mycobacterial culture results (see Table 2). KPH, Kaiser Permanente Hawaii; NHOPI, Native Hawaiian and Other Pacific Islander; NTM, nontuberculous mycobacteria; NTMPD, nontuberculous mycobacterial pulmonary disease; TB, tuberculosis. †Only includes KPH patients residing in a single ZIP code throughout the study period (n = 286,322). ‡Some patients self-reported >1 racial/ethnic category; percentage calculated out of total patients reporting race/ethnicity (n = 292,336).

Compared with the overall KPH population (Table 1), NTM infection patients were significantly older (mean age + SD 66 + 16 years vs. 36 + 22 years; p<0.05), and a greater proportion were female (54% vs. 49%), enrolled in KPH for >5 years (84% vs. 49%), and self-identified as Asian (58% vs. 38%), whereas significantly fewer (p<0.05) were NHOPI (18% vs. 24%) or self-identified as being >1 race/ethnicity (24% vs. 36%). NTMPD patients were similar in age, sex, and racial/ethnic distribution to those with only 1 NTM-positive culture (Table 1). 

Of the 455 patients who had a mycobacterial culture performed, 40 (2%) had positive results for *M. tuberculosis*. TB patients were younger (mean age + SD 55 + 16 years) and a greater proportion were male (n = 28; 70%) compared with the KPH population. Among TB patients, 30 (75%) self-identified as Asian, 5 (13%) as NHOPI, and 4 (10%) as white; 6 (15%) self-identified as >1 race/ethnicity. Of the 40 TB patients, 5 (13%) were co-infected with NTM (all with MAC and 1 additionally with *M. fortuitum* group).

### Prevalence of NTM Isolation

**Figure 2:**
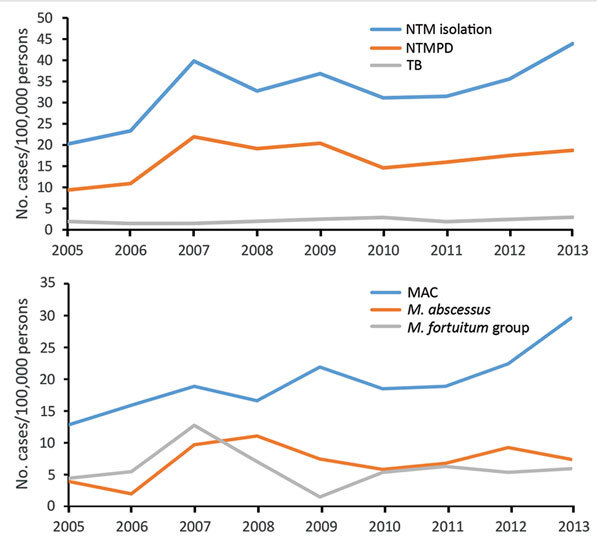
Annual prevalence of pulmonary nontuberculous mycobacteria isolation, nontuberculous mycobacterial pulmonary disease, and tuberculosis (A) and annual prevalence of pulmonary nontuberculous mycobacteria isolation by species (B) among a cohort of Kaiser Permanente Hawaii patients, Hawaii, 2005–2013. MAC, *Mycobacterium avium* complex; NTM, nontuberculous mycobacteria; NTMPD, nontuberculous mycobacterial pulmonary disease; TB, tuberculosis.

The annual prevalence of NTM isolation more than doubled over time, from 20 cases/100,000 persons in 2005 to 44 cases/100,000 persons in 2013 (annual percentage change 6%, 95% CI 1%–11%; p = 0.01). NTMPD prevalence also doubled, from 9 to 19 cases/100,000 persons, although this increase was not significant (p = 0.2) (Figure 2, panel A). When evaluated by species, this trend was observed for MAC only (Figure 2, panel B).

**Figure 3:**
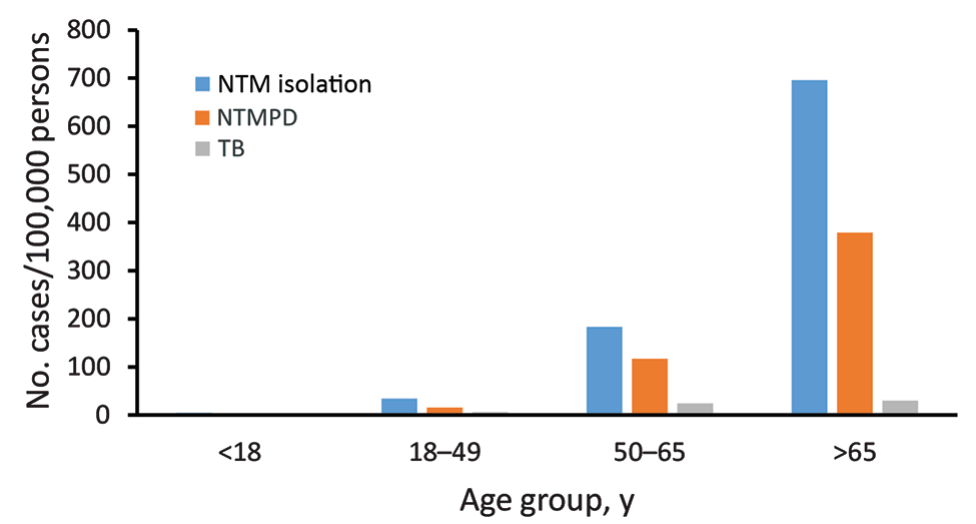
Overall period prevalence of pulmonary nontuberculous mycobacteria isolation, nontuberculous mycobacterial pulmonary disease, and tuberculosis, by age group, among a cohort of Kaiser Permanente Hawaii patients, Hawaii, 2005–2013. NTM, nontuberculous mycobacteria; NTMPD, nontuberculous mycobacterial pulmonary disease; TB, tuberculosis.

The 2005–2013 period prevalence for NTM isolation was 122 cases/100,000 persons. Prevalence was 4-fold greater among persons >65 years of age than for those 50–64 years of age (696 vs. 183 cases/100,000 persons) (Figure 3). For persons >75 years of age, period prevalence was 906 cases/100,000 persons, and within this age group, prevalence was substantially higher among those enrolled in KPH for >5 years (1,049 cases/100,000 persons) compared with those enrolled for only 2–4 years (577 cases/100,000 persons). NTM isolation period prevalence was highest on Oahu (153 cases/100,000 persons), followed by Maui (91 cases/100,000 persons) and Hawaii (the Big Island) (84 cases/100,000 persons); no cases were identified on other islands.

**Figure 4:**
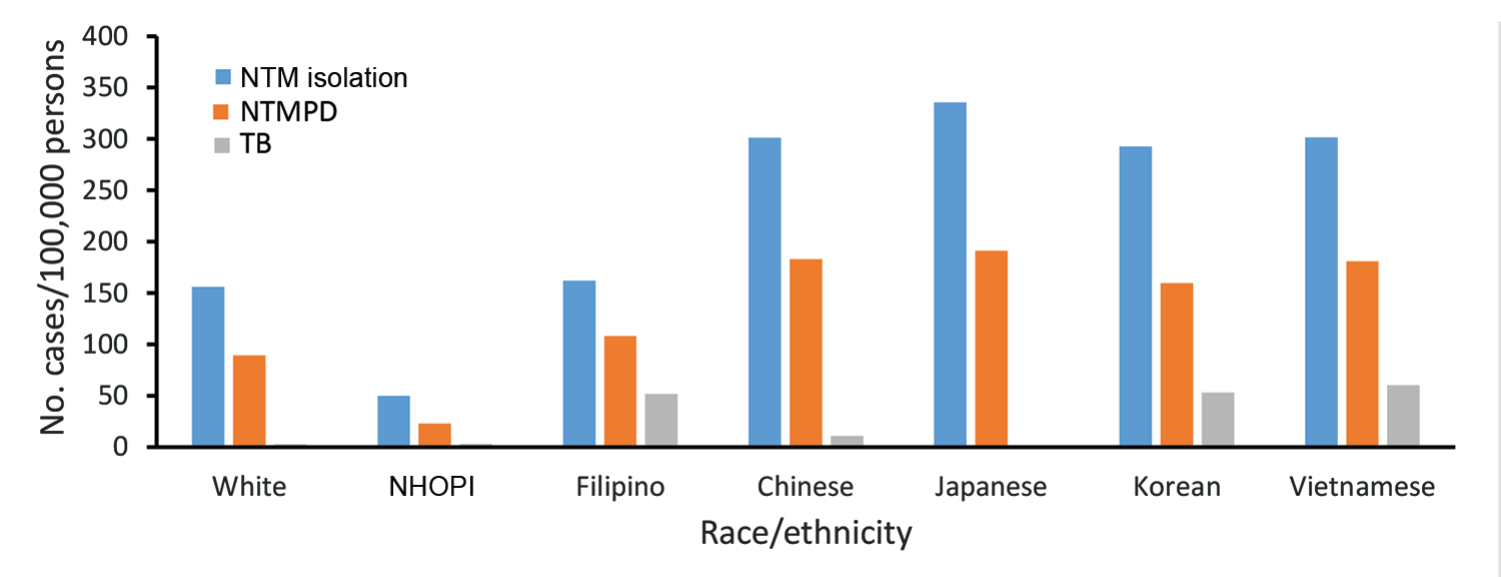
Overall period prevalence of pulmonary nontuberculous mycobacteria isolation, nontuberculous mycobacterial pulmonary disease, and tuberculosis, by race/ethnicity, among a cohort of Kaiser Permanente Hawaii patients, Hawaii, 2005–2013. NHOPI, Native Hawaiians and Other Pacific Islanders; NTM, nontuberculous mycobacteria; NTMPD, nontuberculous mycobacterial pulmonary disease; TB, tuberculosis.

**Figure 5:**
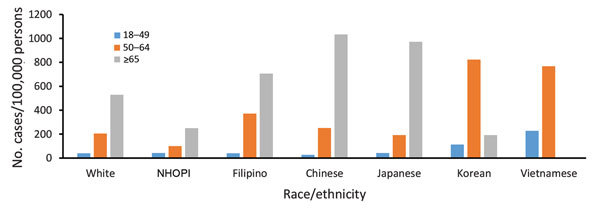
Overall period prevalence of pulmonary nontuberculous mycobacteria isolation, by race/ethnicity and age group, among a cohort of Kaiser Permanente Hawaii patients, Hawaii, 2005–2013. No cases of nontuberculous mycobacteria isolation were reported among Vietnamese patients >65 years of age. NHOPI, Native Hawaiians and Other Pacific Islanders.

**Figure 6:**
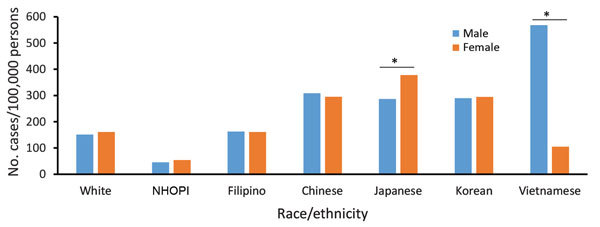
Overall period prevalence of pulmonary nontuberculous mycobacteria isolation, by race/ethnicity and sex, among a cohort of Kaiser Permanente Hawaii patients, Hawaii, 2005–2013. Prevalence reflects number of unique patients with pulmonary nontuberculous mycobacteria detected during the 9-year period. *p<0.05 (significant difference).

NTM isolation period prevalence was highest among Japanese, Chinese, Korean, and Vietnamese patients (≈300 cases/100,000 persons; average annual prevalence 34 cases/100,000 persons), similar among Filipino and white patients (162 and 156 cases/100,000 persons, respectively; average annual prevalence 18 cases/100,000 persons), and lowest among NHOPI patients (50 cases/100,000 persons; average annual prevalence 6 cases/100,000 persons) (Figures 4, 5). NTM isolation prevalence was progressively greater by increasing age group across nearly all racial/ethnic groups evaluated (Figure 5). Among Vietnamese and Korean patients, the highest NTM isolation rates were observed among those 50–64 years of age (767 and 823 cases/100,000 persons, respectively); however, these estimates did not significantly differ from those observed among persons >65 years of age in these populations (p>0.2). Sex differences in NTM isolation prevalence were also noted by racial/ethnic group (Figure 6). Among Vietnamese patients, NTM isolation was more prevalent among men than women (568 vs. 105 cases/100,000 persons), whereas among Japanese patients, NTM isolation was more prevalent among women than men (378 vs. 287 cases/100,000 persons). For all other racial/ethnic groups, prevalence did not differ greatly by sex. NTM isolation prevalence was consistently double the NTMPD prevalence.

### TB Incidence

TB incidence was stable over time (≈2 cases/100,000 persons/year) (Figure 2, panel A). The cumulative 9-year rate was 11 cases/100,000 persons and was higher among men than women (15 vs. 7 cases/100,000 persons). The overall TB rate was highest among those >50 years of age and did not differ between those 50–65 years of age (24 cases/100,000 persons) and those >65 years of age (30 cases/100,000 persons) (Figure 3). TB incidence was highest on Maui (21 cases/100,000 persons), followed by Oahu (11 cases/100,000 persons) and the Big Island (8 cases/100,000 persons).

Among racial/ethnic groups, the TB rate was lowest among Japanese (no cases), white, and NHOPI patients (3 cases/100,000 persons for both) and highest among other patients of Asian ethnicities (33 cases/100,000 persons) (Figure 4). Most (55%) TB patients were Filipino, with an overall incidence of 52 cases/100,000 persons; although only 2 (5%) TB patients were Korean and 1 (3%) Vietnamese, their overall incidence was similarly high (53 and 60 cases/100,000 persons, respectively) (Figure 4).

### Co-morbid Conditions

Of the co-morbidities evaluated (Table 2), the most frequently reported conditions among NTM infection patients were COPD (41%) and bronchiectasis (37%). Bronchiectasis was more frequent among patients with *M. abscessus* infection (58%) than those with MAC (39%) or *M. fortuitum* group (31%) infection, whereas COPD was more frequent among patients with MAC infection (45%) than those with *M. abscessus* (38%) or *M. fortuitum* group (36%) infection. Patients with NTMPD had a similar co-morbidity profile. For TB patients, few co-morbidities were reported, although COPD (13%) was most common. Although only 4 KPH patients with cystic fibrosis were tested for mycobacteria in this cohort, 2 (50%) had NTMPD. 

**Table 2:** Occurrence of selected co-morbid conditions among Kaiser Permanente Hawaii patients who had mycobacterial cultures performed, Hawaii, 2005–2013*

Co-morbidity	No. (%)							
	Total, n = 2,197†	NTM isolated				NTMPD, n = 254	TB, n = 40	Culture negative, n = 1,707
		NTM, n = 455	MAC, n = 290	*M. abscessus*, n = 87	*M. fortuitum*, n = 109			
COPD	780 (36)	188 (41)	130 (45)	33 (38)	39 (36)	108 (43)	5 (13)	588 (34)
Bronchiectasis	335 (15)	169 (37)	114 (39)	50 (58)	34 (31)	113 (44)	1 (3)	165 (10)
Cystic fibrosis	4 (0.2)	2 (0.4)	1 (0.3)	1 (1)	0 (0)	2 (0.8)	0 (0)	2 (0.1)
HIV	24 (1)	4 (0.9)	3 (1)	0 (0)	1 (0.9)	4 (2)	0 (0)	20 (1)
Coccidiomycosis	12 (0.6)	4 (0.9)	2 (0.7)	0 (0)	1 (0.9)	1 (0.4)	0 (0)	8 (0.5)
Sarcoidosis	10 (0.5)	3 (0.7)	2 (0.7)	0 (0)	1 (0.9)	0 (0)	0 (0)	7 (0.4)
Malignant neoplasm of trachea, bronchus, or lung	472 (21)	56 (12)	34 (12)	8 (9)	12 (11)	27 (11)	0 (0)	416 (24)
Other malignancies	453 (21)	92 (20)	62 (21)	16 (18)	20 (18)	52 (20)	5 (13)	357 (21)

*COPD, chronic obstructive pulmonary disease; MAC, *Mycobacterium avium* complex; NTM, nontuberculous mycobacteria; NTMPD, NTM pulmonary disease; TB, tuberculosis. †Total number and percentage of patients with co-morbidity out of all Kaiser Permanente Hawaii patients with pulmonary mycobacterial culture results.

### Species-Specific Risk Analysis

We obtained logistic regression results by mycobacterial species adjusted for sex, age, and years in KPH (Table 3). Compared with all other KPH patients, NHOPI patients were at decreased risk for NTM infection (aOR 0.5, 95% CI 0.3–0.9), whereas an increased risk for NTM infection and TB was observed among Asian patients, particularly those who were Vietnamese (NTM, aOR 3.7, 95% CI 1.6–8.6; TB, aOR 9.6, 95% CI 2.0–46.8), Korean (NTM, aOR 1.9, 95% CI 1.1–3.4; TB, aOR 5.9, 95% CI 1.7–20.4), and Filipino (NTM, aOR 1.3, 95% CI 1.3, 1.0–1.7; TB, aOR 8.8, 95% CI 4.9–16.0). However, differences in risk were noted by NTM species (Table 3). Japanese patients were nearly twice as likely to have *M. abscessus* infection (aOR 2.0, 95% CI 1.2–3.2) but were not at increased risk for MAC or *M. fortuitum* group infection compared with other racial/ethnic groups. Filipino patients also were at increased risk for *M. abscessus* (aOR 2.0, 95% CI 1.2–3.3) and MAC (aOR 1.5, 95% CI 1.1–2.1) infection. Vietnamese patients were more likely to have MAC (aOR 3.7, 95% CI 1.3–10.6), *M. fortuitum* group (aOR 8.7, 95% CI 3.0–25.0), and *M. abscessus* (aOR 5.0, 95% CI 1.0–24.6) infection. Korean patients were only more likely to have *M. fortuitum* group infection (aOR 4.0, 95% CI 1.7–9.5). A substantially higher risk for co-infections with multiple NTM species was observed for only Vietnamese patients (aOR 17.3, 95% CI 5.9–50.6).

**Table 3:** Risk for nontuberculous mycobacterial pulmonary disease (by mycobacteria species) or tuberculosis among Kaiser Permanente Hawaii patients, by demographic characteristics and co-morbid condition, Hawaii, 2005–2013*

Characteristic	aOR (95% CI)				
	NTM	*M. abscessus*	MAC	*M. fortuitum* group	TB
Racial/ethnicity					
White	0.9 (0.7–1.1)	0.7 (0.4–1.1)	1.0 (0.7–1.2)	1.0 (0.6–1.5)	**0.2 (0.05–0.6)**
NHOPI	**0.5 (0.3–0.9)**	0.1 (0.01–1.4)	**0.4 (0.2–0.9)**	0.5 (0.2–1.3)	0.5 (0.1–2.6)
Black	0.6 (0.1–3.1)	1.1 (0.7–16.4)	1.0 (0.2–4.8)	0.7 (0.05–11.5)	NA
Asian	**1.4 (1.2–1.7)**	**2.5 (1.7–3.9)**	**1.4 (1.1–1.8)**	1.5 (1.0–2.2)	**4.9 (2.6–9.2)**
Filipino	**1.3 (1.0–1.7)**	**2.0 (1.2–3.3)**	**1.5 (1.1–2.1)**	1.3 (0.7–2.1)	**8.8 (4.9–16.0)**
Japanese	1.2 (1.0–1.6)	**2.0 (1.2–3.2)**	1.0 (0.7–1.4)	**1.6 (1.0–2.5)**	NA
Chinese	1.3 (0.9–2.0)	1.9 (0.9–3.9)	1.5 (0.95–2.3)	0.3 (0.06–1.5)	1.0 (0.2–5.0)
Korean	**1.9 (1.1–3.4)**	2.0 (0.6–7.0)	1.4 (0.6–3.2)	**4.0 (1.7–9.5)**	**5.9 (1.7–20.4)**
Vietnamese	**3.7 (1.6–8.6)**	**5.0 (1.0–24.6)**	**3.7 (1.3–10.6)**	**8.7 (3.0–25.0)**	**9.6 (2.0–46.8)**
Island of residence					
Oahu	**1.5 (1.2–1.9)**	**2.6 (1.4–5.2)**	**1.6 (1.2–2.2)**	1.3 (0.8–2.0)	0.6 (0.3–1.2)
Maui	**0.7 (0.6–1.0)**	**0.4 (0.2–0.9)**	0.7 (0.5–1.0)	0.8 (0.5–1.4)	**2.3 (1.2–4.3)**
Hawaii	**0.6 (0.4–1.0)**	0.5 (0.2–1.4)	0.6 (0.4–1.1)	0.9 (0.5–2.0)	0.7 (0.2–2.3)
Age group, y					
<18	Referent	Referent	Referent	Referent	Referent
18–49	**7.4 (2.9–19.3)**	1.9 (0.3–11.3)	**7.8 (2.2–27.9)**	**8.3 (1.6–42.7)**	3.9 (0.7–21.0)
50–64	**31.2 (12.2–79.7)**	**22.1 (4.4–111.8)**	**32.7 (9.3–114.6)**	**26.8 (5.3–135.5)**	**13.4 (2.6–69.1)**
>65	**106.4 (42.0–270.0)**	**65.1 (13.0–325.1)**	**127.1 (36.6–441.3)**	**61.4 (12.2–308.1)**	**16.6 (3.1–88.5)**
Sex					
M	Referent	Referent	Referent	Referent	Referent
F	1.0 (0.9–1.3)	1.4 (0.9–2.1)	1.1 (0.9–1.4)	0.8 (0.6–1.2)	**0.4 (0.2–0.8)**
Years in KPH					
1	Referent	Referent	Referent	Referent	Referent
2–4	**2.6 (1.4–4.7)**	1.1 (0.4–2.9)	**2.7 (1.1–6.3)**	**7.0 (1.3–36.3)**	1.7 (0.5–5.7)
>5	**6.4 (3.6–11.2)**	2.3 (0.96–5.4)	**7.7 (3.5–16.8)**	**14.9 (3.0–74.2)**	2.3 (0.8–6.9)
Co-morbid condition†					
Bronchiectasis	**8.3 (6.5–10.7)**	**12.0 (7.6–18.8)**	**7.0 (5.2–9.2)**	**4.6 (2.9–7.1)**	0.4 (0.09–2.2)
COPD	**1.8 (1.4–2.2)**	1.3 (0.8–2.0)	**1.9 (1.5–2.5)**	1.5 (1.0–2.3)	0.4 (0.2–1.1)
Coccidiomycosis	**4.5 (1.4–15.1)**	2.0 (0.1–38.4)	3.5 (0.8–15.2)	4.0 (0.7–23.4)	2.8 (0.2–50.1)
Sarcoidosis	2.0 (0.6–7.2)	1.2 (0.1–22.8)	2.2 (0.5–9.4)	3.5 (0.6–20.0)	3.0 (0.2–54.6)
Malignant neoplasm‡	**0.7 (0.5–0.9)**	**0.5 (0.2–1.0)**	0.6 (0.4–0.9)	0.7 (0.4–1.3)	**0.07 (0.01–1.0)**
Other malignancies	**0.7 (0.5–0.8)**	**0.5 (0.3–0.8)**	**0.7 (0.5–0.9)**	0.7 (0.5–1.1)	**0.3 (0.1–0.8)**

*aOR, adjusted odds ratio; COPD, chronic obstructive pulmonary disease; KPH, Kaiser Permanente Hawaii; MAC, *Mycobacterium avium* complex; NHOPI, Native Hawaiian and Other Pacific Islander; NTM, nontuberculous mycobacteria; Ref, referent group; TB, tuberculosis; NA, not available because limited sample size resulted in unstable model estimates. All aORs and 95% CIs associated with each racial/ethnic group, island, and co-morbid condition were assessed independently as a binary variable via logistic regression models, adjusted for age, sex, and years in KPH. Estimates for years in KPH, age, and sex were obtained from a single adjusted multivariable model. Race/ethnicity was self-reported; race/ethnicity-specific analyses were limited to patients reporting only 1 racial/ethnic group. Statistical significance (p<0.05) is indicated with boldface font.  †Data only available for KPH patients with a mycobacterial culture performed; percentages listed are out of total patient population with that co-morbidity. ‡Of trachea, bronchus, or lung.

NTM infection patients were more likely to have bronchiectasis, whereas only patients with MAC infection were additionally more likely to have COPD (p<0.001) (Table 3). After also adjusting for COPD, Asian patients remained at increased risk for NTM infection (aOR 1.4, 95% CI 1.2–1.7), whereas white patients were at decreased risk (aOR 0.8, 95% CI 0.6–1.0). Similarly, Japanese (aOR 1.9, 95% CI 1.1–3.0) and Filipino (aOR 1.7, 95% CI 1.0–2.9) patients remained at increased risk for *M. abscessus* infection, as did Vietnamese (aOR 6.0, 95% CI 1.8–19.6) and Korean (aOR 3.9, 95% CI 1.6–9.7) patients for *M. fortuitum* group infection. After controlling for COPD status, Japanese patients were no longer more likely to have *M. fortuitum* group infection, and only Filipino patients (aOR 1.4, 95% CI 1.0–2.0) remained at increased risk for MAC infection.

## Discussion

We identified significant epidemiologic trends and species-specific differences in the prevalence of NTM infection in Hawaii. By using patient laboratory data from a representative population of Hawaii residents enrolled in a closed healthcare system, we found that the prevalence of NTM infection was double that previously reported (*1*). However, epidemiologic differences by species and race/ethnicity were noted within Hawaii.

Among persons living in Hawaii, Asians are at greater risk for both NTM infection and TB compared with other racial/ethnic groups, although this varied by racial/ethnic subgroup and mycobacterial species. Japanese patients were at increased risk for *M. abscessus* infection only, whereas Vietnamese and Korean patients were at a substantially higher risk for both *M. fortuitum* group infection and TB compared with others in Hawaii. Filipino patients were at increased risk for *M. abscessus* infection, MAC infection, and TB. In contrast, NHOPI patients were less likely to have NTM infection than all others. Nonetheless, all non-Asian populations evaluated, including whites and NHOPIs, had a higher estimated NTMPD prevalence than has been reported elsewhere in the country (*1*,*2*,*18**–**20*).

The increased prevalence of NTM infection in Hawaii might be attributable to unique environmental conditions. Soils are high in humic acid, a component associated with higher numbers of mycobacteria (*14*,*21**–**23*), which might contribute to an increased potential for environmental exposure through not just soil but also water sources, because soil is often the source of waterborne pathogens (*24*), and both can result in the generation of bioaerosols that might contain mycobacteria (*25*). Our previous studies identified factors related to a greater persistence of moisture droplets in the air, including higher saturated vapor pressure and evapotranspiration rates, to be associated with a greater risk for NTM infection (*14**,**26*). Additional, systematic environmental sampling is needed to speculate further on exposure sources.

Higher rates of NTM infection have been reported in East Asian populations, although global studies are limited (*27*). In 2005, the annual prevalence of NTM infection in Japan was estimated to be 33–65 cases/100,000 persons (*28*), which is similar to the rate identified in our present study. NTM infection was also frequently reported among hospital patients in South Korea (*29*). However, a study from Taiwan estimated an annual prevalence of NTM infection of 8 cases/100,000 persons in 2008 (*30*), which is markedly less than what we observed in Hawaii. Assessing the role of race/ethnicity in mycobacterial lung disease is complex because these categorizations likely reflect behavioral, cultural, and biologic factors (*31*).

An increasing trend was detected only for MAC infection; infection rates with all other NTM species remained relatively stable over time. A similar increasing trend in NTM infection prevalence was reported among US Medicare patients, although in that study species-level data were unavailable (*1*). It is impossible to know if this rise in prevalence is attributable to greater environmental exposure or host susceptibility or is in part attributable to greater clinical awareness and detection; however, the latter seems unlikely given that an increase was only observed for 1 species.

In contrast to NTM, annual rates of TB were substantially lower than what has been reported for Hawaii (*12*). This finding is likely attributable to differences in risk among the KPH population compared with all Hawaiians with TB. The difference was especially notable for NHOPIs (*32*), who in this population had TB incidence rates similar to that of whites. However, even among KPH patients, the incidence of TB was 5 times higher among Vietnamese, Korean, and Filipino patients compared with the overall population.

Different co-morbidity patterns were observed by mycobacteria species. Although all NTM infection patients were more likely to have bronchiectasis regardless of species, only patients with MAC infection were additionally more likely to have COPD. After controlling for COPD status along with other factors in models, the association between MAC infection and Vietnamese race/ethnicity was no longer significant, potentially reflecting behavioral contributions to their increased risk (assuming COPD is a proxy for smoking status) (*33*). In fact, Vietnamese and Korean NTM infection patients were similar to TB patients in terms of their age and sex, with a higher proportion of middle-aged persons affected and, for Vietnamese patients, more male than female patients. However, estimates from persons >65 years of age were limited for Vietnamese and Korean patients because of smaller population sizes, making lower observed rates compared with those aged 50–65 possibly an artifact of fewer opportunities to detect NTM. Japanese NTM infection patients, on the other hand, were more likely to be female, older, and have *M. abscessus* infection compared with other racial/ethnic groups. Previous studies have reported racial/ethnic disparities in smoking rates in Hawaii, with Japanese and Chinese persons reporting significantly lower rates of nicotine dependence than other Asian groups, which might explain some of the trends and associations we observed (*34*).

Differences in prevalence were also noted by island, with NTM infection patients more likely to reside on Oahu. NTM environmental exposure levels may vary by island, perhaps because of differences in island ecology or variations in water sources and distribution systems. Differences in exposure might also be related to factors associated with residing in a more urban area such as Honolulu. However, even within the KPH system, patients living in closer proximity to more specialized healthcare facilities are probably more likely to be tested for mycobacteria, given symptoms. Similarly, even after controlling for age, longer enrollment time in KPH was associated with a substantially higher risk for NTM infection. The association with enrollment time could be a proxy for increased duration of exposure to NTM in Hawaii, but it could also reflect greater access to healthcare over a longer period of time. Regardless of whether this increased risk for NTM infection is attributable to greater environmental exposure or just increased access to care, the dose-response effect observed between KPH enrollment time and NTMPD risk demonstrates that the prevalence estimates we generated probably reflect underestimates of the actual burden of NTM infection in Hawaii, given that half the patients were enrolled for <4 years.

This study is subject to several limitations. These findings reflect the epidemiology of mycobacterial infections in patients participating in the KPH system and might not be representative of those without access to similar healthcare plans, who likely differ socioeconomically. Although income data on KPH patients were unavailable, previous studies have shown higher prevalence of NTM infection in higher-income areas, likely because of greater access to diagnostic and clinical services (*14*). Similarly, because most KPH patients reside in Oahu, we might be unable to generalize these findings to other islands, especially those with limited KPH representation. Additional studies are needed to better identify interisland differences in mycobacterial infection risk. We were also unable to assess actual time of exposure in Hawaii because data on length of time residing in Hawaii were unavailable. Additionally, co-morbidity data were based on ICD-9 codes; therefore, actual co-morbidity status might have been misclassified for some patients. Last, some patients classified as negative in our models might have been in fact positive for NTM infection but were not tested and identified in our dataset; however, because the NTM infection is rare, this would probably affect very few patients and have limited effect on our results. Despite these limitations, differences in prevalence by race/ethnicity were striking, underscoring the importance of genetic, environmental, and behavioral contributions to risk for NTM infection. 

In conclusion, we identified differences in the epidemiology of pulmonary mycobacterial infection and disease in Hawaii residents by racial/ethnic group. The prevalence of NTM infection and TB are far greater in Hawaii than elsewhere in the United States, probably because of a combination of increased environmental exposure and a possibly more susceptible population, attributable in part to its unique demographic profile. In particular, Asians living in Hawaii are at significantly higher risk for NTM infection and TB compared with other racial/ethnic groups, whereas NHOPIs appear to be at decreased risk compared with all others. Additional prospective studies assessing genetic, behavioral, and environmental risk factors in high-risk regions such as Hawaii are needed to better understand the role of race/ethnicity in mycobacterial lung disease.
